# Mutator Phenotype and DNA Double-Strand Break Repair in BLM Helicase-Deficient Human Cells

**DOI:** 10.1128/MCB.00443-16

**Published:** 2016-11-14

**Authors:** Tetsuya Suzuki, Manabu Yasui, Masamitsu Honma

**Affiliations:** Division of Genetics and Mutagenesis, National Institute of Health Sciences, Setagaya-ku, Tokyo, Japan

## Abstract

Bloom syndrome (BS), an autosomal recessive disorder of the *BLM* gene, predisposes sufferers to various cancers. To investigate the mutator phenotype and genetic consequences of DNA double-strand breaks (DSBs) in BS cells, we developed BLM helicase-deficient human cells by disrupting the *BLM* gene. Cells with a loss of heterozygosity (LOH) due to homologous recombination (HR) or nonhomologous end joining (NHEJ) can be restored with or without site-directed DSB induction. BLM cells exhibited a high frequency of spontaneous interallelic HR with crossover, but noncrossover events with long-tract gene conversions also occurred. Despite the highly interallelic HR events, BLM cells predominantly produced hemizygous LOH by spontaneous deletion. These phenotypes manifested during repair of DSBs. Both NHEJ and HR appropriately repaired DSBs in BLM cells, resulting in hemizygous and homozygous LOHs, respectively. However, the magnitude of the LOH was exacerbated in BLM cells, as evidenced by large deletions and long-tract gene conversions with crossover. BLM helicase suppresses the elongation of branch migration and crossover of double Holliday junctions (HJs) during HR repair, and a deficiency in this enzyme causes collapse, abnormal elongation, and/or preferable resolution to crossover of double HJs, resulting in a large-scale LOH. This mechanism underlies the predisposition for cancer in BS.

## INTRODUCTION

DNA double-strand breaks (DSBs) are the most dangerous form of DNA damage (1). DSBs are caused by ionizing radiation or radiometric chemicals, but they can also occur spontaneously during DNA replication. Other DNA damage, such as single-strand breaks, can easily be converted to DSBs via interaction with replication forks (2). DSBs are generally repaired through nonhomologous end joining (NHEJ) or homologous recombination (HR) (3–5). NHEJ connects the broken ends of sequences with little or no homology in a nonconservative manner, and some genetic information is lost during this process (6). Conversely, HR requires extensive tracts of sequence homology and is essentially error free (7, 8). HR is the primary DSB repair pathway in yeasts and prokaryotes; however, NHEJ has been suggested as the primary pathway in mammalian cells (9). Regardless, both HR and NHEJ are important for maintaining genomic integrity in mammalian cells, and defects in either of these pathways can lead to cell death or neoplastic transformation.

**FIG 1:**
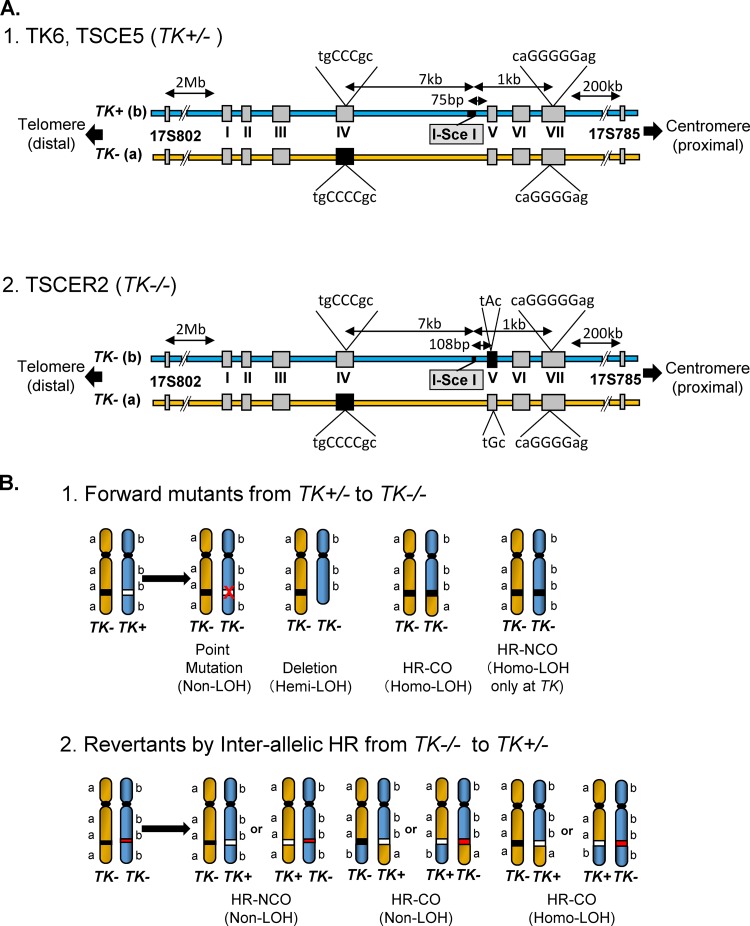
(A) Schematic of the thymidine kinase gene (*TK*) locus in TK6 cells and their derivatives. The *TK^+^* and *TK^−^* alleles in TK6 cells are the functional and nonfunctional forms, respectively. The *TK^−^* (a) allele harbors a frameshift mutation (CCC to CCCC) in exon 4 that inactivates TK function. The *TK^−^* (a) allele also has a frameshift mutation (GGGGG to GGGG) in exon 7 that does not affect TK function. TK6 cells have polymorphic microsatellite markers on the proximal (17S785) and distal (17S802) sides of the *TK* locus. TSCE5 cells have a 31-bp DNA fragment containing the 18-bp I-SceI site inserted 75 bp upstream of exon 5. TSCER2 cells have an additional point mutation (G-to-A transition) at bp 23 of exon 5. Thus, TSCER2 cells are compound heterozygotes (*TK*^−/−^) for the *TK* gene. The black box indicates the mutated exons. (B) Models of the mechanisms that generate TK mutants and TK revertants from *TK*-heterozygous (*TK*^+/−^) and *TK*-compound-heterozygous (*TK*^−/−^) cells, respectively. TK6 and TSCE5 cells carry functional (b; *TK^+^*) and nonfunctional (a; *TK^−^*) *TK* alleles. Inactivation of the *TK^+^* allele by a point mutation, deletion, or interallelic homologous recombination (HR), with crossover (CO) or no crossover (NCO) between the alleles, causes TK-deficient mutants. Large deletions and interallelic HR are expressed as a loss of heterozygosity (LOH). TSCER2 cells are compound heterozygotes (*TK*^−/−^) for the *TK* gene. Interallelic HR between the *TK^−^* alleles generates the *TK^+^* allele, thus producing TK-proficient revertants. The revertants showed LOH or non-LOH depending on the mechanism employed (CO or NCO). The white box indicates the *TK^+^* allele, and the black and red boxes indicate the *TK^−^* allele.

Loss of heterozygosity (LOH) in tumor suppressor genes is a critical event during the development of human cancers (10, 11). LOH can result from a large deletion caused by NHEJ or interallelic HR during DSB repair (12, 13). Similar LOH-type genetic alterations are observed in autosomal recessive gene mutation assays, which are useful tools for the elucidation of DSB repair mechanisms. The thymidine kinase gene (*TK*) mutation assay uses *TK*-heterozygous TK6 cells (*TK*^*+/*−^) to detect a wide mutational spectrum, with mutations ranging from point mutations to LOH-type mutations (14). Large deletions caused by NHEJ result in hemizygous LOH (hemi-LOH), and interallelic HR leads to homozygous LOH (homo-LOH) (Fig. 1B, panel 1). In contrast, compound-heterozygous *TK*^−*/*−^ TK6 cells, which have biallelic mutations in exons 4 and 5 of the *TK* gene (Fig. 1A, panel 2), can produce TK-proficient revertants (*TK*^*+/*−^) when DSBs are repaired by interallelic HR (Fig. 1B, panel 2). Thus, a positive-negative selection of TK phenotypes and LOH analysis using the TK6 cell assay system can elucidate NHEJ and HR repair mechanisms.

Based on this assay system, we successfully introduced a meganuclease I-SceI site into the *TK* gene (15). TSCE5 and TSCER2 cells are *TK*-heterozygous (*TK*^+/−^) and compound-heterozygous (*TK*^−/−^) cells, respectively, and both of these cell types have an I-SceI site in intron 4 of the *TK* gene (Fig. 1A). DSBs can be generated at the I-SceI site by the introduction of an I-SceI enzyme expression vector. This system enables the tracking of a DSB after NHEJ or HR. Indeed, we previously demonstrated that I-SceI-induced DSBs in human cells were predominantly repaired by NHEJ, which resulted in small deletions; furthermore, repairs via HR were minimal, even during the late S/G_2_ phases (16, 17). Notably, cells deficient in DNA repair, especially NHEJ and HR, may exhibit different consequences.

BLM helicase is an important regulator of HR (18, 19) and is a member of the RecQ family of DNA helicases. To date, five DNA helicases of the RecQ family (BLM, WRN, RecQL, RecQ4, and RecQ5) have been identified in humans (18). Mutations in *BLM*, *WRN*, and *RecQ4* cause Bloom syndrome (BS), Werner syndrome, and Rothmund-Thomson syndrome, respectively (20, 21). Patients with BS display growth retardation, immunodeficiency, sunlight sensitivity, and cancer predisposition from a young age (21). In addition to these clinical manifestations, BS cells exhibit a number of cytogenetic abnormalities, including chromosome breakage, quadriradial chromatid interchanges, and an especially high degree of sister chromatid exchange (SCE), which is the hallmark of BS (22–24). Numerous studies on the molecular mechanisms of BLM helicase involved in genomic instability and cancer predisposition have been published previously. BLM helicase is involved in multiple cellular processes that require HR repair, telomere maintenance, and replication (18, 19).

There are multiple aspects of DSB repair by HR. Broken DNA ends adjacent to the DSB are resected to yield 3′ overhangs of single-stranded DNA (ssDNA). The exposed ssDNA is rapidly coated by the ssDNA binding protein RPA, which is then displaced by RAD51 in a process that requires RAD52 and several other mediators. The RAD51 nucleofilament mediates homology searching and strand invasion at a homologous template, thus forming a displacement loop. After second-end capture, the recombination intermediates, known as double Holliday junctions (HJs), form and subsequently complete the repair process (7, 8). BLM helicase has multiple roles in the early and late stages of HR regulation. In the early stage, BLM helicase binds to DNA2 and promotes the extensive resection that occurs after the MRE11-mediated initial resection of the DNA ends at the DSB (25). In later stages, BLM helicase is involved in branch migration and the resolution of double HJs (18, 19). The increase of SCEs in BS cells is thought to be due to a deficiency in BLM helicase during the resolution of HJs, thus underlining the importance of this enzyme. Indeed, a deficiency of BLM helicase is known to increase the LOH (26–28). It is possible to generate LOH via multiple mechanisms, and pro- and anti-HR functions of BLM helicase have been proposed (29, 30).

In this study, we developed BLM helicase-deficient TSCE5 and TSCER2 cells by using zinc finger nuclease (ZFN) technology. Our cytogenetic analysis confirmed that these cells exhibited representative characteristics of BS cells, and we investigated the mutator phenotype and genetic consequences of a DSB in the I-SceI site. We conducted four parallel studies: (i) measurement of the frequency of spontaneously occurring TK mutants and analysis of the LOH spectrum in TSCE5 cells, (ii) measurement of the frequency of spontaneously occurring TK revertants and analysis of the LOH spectrum in TSCER2 cells, (iii) measurement of the frequency of TK mutants and analysis of the LOH spectrum in TSCE5 cells following induction of site-directed DSBs, and (iv) measurement of the frequency of TK revertants and analysis of the LOH spectrum in TSCER2 cells following induction of site-directed DSBs. BLM helicase-deficient cells exhibited a mutator phenotype with highly spontaneous mutant and revertant frequencies (MF and RF, respectively) and showed large-scale LOHs after DSB repair by NHEJ and HR. These results may constitute the fundamental mechanism underlying the cancer predisposition phenotype of BS. Based on the role of BLM helicase in HR and DSB repair, we propose that the mutator phenotype and genomic instability caused by BLM helicase deficiency result in large-scale LOHs.

## MATERIALS AND METHODS

### Cells and cell culture.

We used the human lymphoblastoid cell lines TSCE5 and TSCER2, which are TK6 cells with an I-SceI site inserted into the *TK* gene (15). TSCE5 cells are TK proficient (*TK*^+/−^), and TSCER2 cells are TK deficient (*TK*^−/−^) (Fig. 1A). Cells were grown in RPMI 1640 medium (Nacalai Tesque, Inc., Kyoto, Japan) supplemented with 10% heat-inactivated horse serum (JRH Biosciences, Lenexa, KS), 200 μg/ml sodium pyruvate, 100 U/ml penicillin, and 100 μg/ml streptomycin and were maintained at 10^5^ to 10^6^ cells/ml at 37°C in a 5% CO_2_ atmosphere with 100% humidity.

### Creation of BLM helicase-deficient cells.

We purchased a CompoZr Knockout ZFN kit for the human *BLM* gene (NM_000057) from Sigma-Aldrich Japan K.K. (Tokyo, Japan). ZFN1 and ZFN2 plasmids that target the human *BLM* gene were supplied in this kit. The target sequence (see Fig. S1A in the supplemental material) was in exon 14 of the human *BLM* gene, which contains the coding sequence for the helicase domain. The ZFN1 and ZFN2 plasmids (25 μg each) were cotransfected into TSCE5 or TSCER2 cells (5 × 10^6^) suspended in 0.1 ml of Nucleofector solution V (Lonza Japan Ltd., Tokyo, Japan) by use of a Nucleofector I device (Lonza Japan Ltd.) according to the manufacturer's instructions. Subsequently, cells were seeded into 96-microwell plates at a density of 0.8 cell/well. After 10 to 14 days, single colonies were independently expanded, and genomic DNA was extracted. A region of exon 14 of the human *BLM* gene was amplified from the genomic DNA by PCR with the following primers: Fw, 5′-TGAGAGGAAGCTCTTGGCACGTT-3′; and Rv, 5′-GGGGGAAATGACAAAGCAGGGTCA-3′. The PCR products were analyzed by agarose gel electrophoresis and DNA sequencing.

### Western blotting.

Cell extracts were prepared for Western blotting by using Pro-Prep (iNtRON Biotechnology, Inc., Cosmo Bio Co., Ltd., Tokyo, Japan). A BLM helicase antibody (sc-7790; Santa Cruz Biotechnology, Inc., Japan) was used as the primary antibody, and a horseradish peroxidase (HRP)-conjugated anti-goat IgG antibody (sc-2020; Santa Cruz Biotechnology, Inc.) was used as the secondary antibody. The HRP signal was detected using an ECL Plus Western blotting detection system (GE Healthcare Bio-Sciences K.K., Tokyo, Japan).

### Cytogenetic analysis of BLM-deficient cells.

For the SCE assay, cultures were incubated with 20 mM 5-bromodeoxyuridine for 28 h in the dark. Colcemid (0.2 mg/ml) was added 2 h prior to harvest of mitotic cells. Metaphase preparation and SCE analysis were performed as previously described (31). Fifty randomly selected metaphase cells were scored to determine the SCE frequency for each cell line. Fisher's exact test was used to statistically analyze the difference in SCE frequency between wild-type and BLM-deficient cells.

The micronucleus (MN) assay was performed as previously described (32). Briefly, cells suspended in a hypotonic KCl solution were incubated for 10 min at room temperature, fixed twice with ice-cold fixative (1:3 glacial acetic acid-methanol), and then resuspended in methanol with 1% acetic acid. A drop of the suspension was placed on a clean glass slide and air dried. The cells were stained with a 40-μg/ml acridine orange solution and immediately observed with an Olympus BX50 fluorescence microscope equipped with a U-MWBV band-pass filter (Olympus Corp., Tokyo, Japan). At least 1,000 intact interphase cells were scored to determine the MN frequency for each cell line. Dunnett's test was used to statistically analyze the difference in MN frequency between wild-type and BLM-deficient cells.

Spectrum karyotyping (SKY) was performed as previously described (33). The probe mixture SkyPaint, containing 24 differentially labeled chromosome-specific painting probes, and Cot-1 blocking DNA (Applied Spectral Imaging Ltd., Tokyo Instruments, Inc., Tokyo, Japan) were used. Metaphase images were captured and analyzed on a SKY Vision cytogenetic workstation (Applied Spectral Imaging Ltd.) attached to an Olympus 50 fluorescence microscope (Olympus Corp.). At least five metaphase cells were analyzed per slide.

### Assessment of drug-resistant thymidine kinase mutants and revertants spontaneously generated or induced by a double-strand break.

The human lymphoblastoid cell lines TSCE5 and TSCER2 were previously created from TK6 cells (15), which have a +1 frameshift mutation in exon 4 of one of the *TK* alleles (“a” allele), resulting in the *TK*-heterozygous genotype (*TK*^+/−^) (Fig. 1A). TSCE5 cells have a 31-bp DNA fragment containing the 18-bp I-SceI site inserted 75 bp upstream of exon 5 of the *TK*^+^ allele (“b” allele) and retain TK function (Fig. 1A). TSCER2 cells have an additional point mutation (G-to-A transition) at base pair 23 of exon 5 of the *TK^+^* allele (“b” allele), resulting in compound heterozygosity (*TK*^−/−^) for the *TK* gene (Fig. 1B). To introduce a DSB into the I-SceI site, an I-SceI expression vector (pCBASce; 50 μg) was transfected into TSCE5 and TSCER2 cells (5 × 10^6^) suspended in 0.1 ml of Nucleofector solution V by use of a Nucleofector I device according to the manufacturer's instructions. Three days later, spontaneous TK mutants or TK revertants (nontransfection) and DSB-induced TK mutants or TK revertants (transfection) were assessed as previously described (17). TSCE5 cells were seeded into 96-microwell plates in the presence of 3 μg/ml trifluorothymidine (TFT) to isolate TK-deficient mutants, and TSCER2 cells were seeded into 96-microwell plates in the presence of 200 μM hypoxanthine, 0.1 μM aminopterin, and 17.5 μM thymidine to isolate TK-proficient revertants. We counted the drug-resistant colonies 2 or 3 weeks later and calculated the mutant frequency (MF) and revertant frequency (RF) according to the Poisson distribution (34). The Student *t* test was used to statistically analyze the differences in MF and RF between wild-type and BLM-deficient cells.

### Molecular analyses of drug-resistant thymidine kinase mutants or revertants spontaneously generated or induced by a double-strand break.

Genomic DNAs from TK mutants and TK revertants were extracted for use as templates in our molecular analyses. We conducted a PCR-based LOH analysis of the human *TK* gene as previously described (35), with slight modifications. Primer sets were used to amplify the portions of exons 4 and 7 of the *TK* gene that are heterozygous for frameshift mutations. Another primer set was used to amplify a portion of β-globin as an internal control. Multiple PCRs with a small number of cycles were performed to coamplify these three regions and quantify the PCR products. The primers used to amplify seven polymorphic microsatellite sequences on chromosome 17q were obtained from the NCBI Gene database (http://www.ncbi.nlm.nih.gov/gene/). To determine the extent of LOH on chromosome 17 and the copy number of the remaining allele, all PCR products were analyzed with an ABI 3130 Avant genetic analyzer and GeneScan and GenoTyper software (Applied Biosystems Japan Ltd., Tokyo, Japan) according to the manufacturer's instructions.

### Allelotype analysis of non-LOH revertants.

Non-LOH revertants from TSCER2 cells were generated by HR with crossover (CO) or no crossover (NCO) (see Fig. 5). CO and NCO cannot be distinguished by standard LOH analysis, because reciprocal interallelic exchange by HR-CO maintains heterozygosity. To differentiate between CO and NCO, TK mutant clones from non-LOH revertants were isolated in the presence of TFT and subjected to LOH analysis of the distal end (36). If the homo-LOH mutant had two “a” alleles, the original LOH revertant was generated by NCO, whereas if the mutant had two “b” alleles, the original LOH revertant was generated by CO (see Fig. S3 in the supplemental material).

### Molecular analyses of non-drug-selected mutants induced by a double-strand break.

After transfection with the I-SceI expression vector (pCBASce; 50 μg), TSCE5 cells were immediately seeded into 96-microwell plates at 1 cell/well in normal culture medium. After 10 days, single colonies were independently expanded, and their genomic DNAs were extracted. A region of the *TK* gene encompassing the I-SceI site was amplified from genomic DNA by PCR with the following primers: Fw, 5′-GCTCTTAAGGAAAAGGAAACAGG-3′; and Rv, 5′-CTGATTCACAAGCACTGAAG-3′. The PCR products were digested with the I-SceI enzyme and analyzed by agarose gel electrophoresis and DNA sequencing (15).

## RESULTS

### Development of BLM-deficient cells by use of zinc finger nuclease technology.

To develop a human cell-based model for studying the role of the BLM helicase, we disrupted the *BLM* gene in human TSCE5 and TSCER2 cells by using ZFN technology. The ZFN plasmids were designed to cleave exon 14 of the *BLM* gene, which contains the coding sequence for the helicase domain (see Fig. S1A in the supplemental material). Because the *BLM* gene is located on chromosome 15 in human cells, biallelic disruption is necessary to develop completely BLM-deficient cells. After transfecting ZFN plasmids into TSCER2 cells, we plated the cells into 96-well plates. After 10 days, we randomly isolated 158 colonies, extracted their genomic DNAs, amplified exon 14 of the *BLM* gene by PCR, and analyzed the PCR products by gel electrophoresis and DNA sequencing. Only 5 of the 158 clones had a deletion in one of the two alleles, and none of the clones had deletions in both alleles. Because BLM-deficient cells were expected to grow more slowly than wild-type cells, we isolated another 16 colonies that appeared 14 days after transfection. Two of these 16 clones had deletions in both alleles. Similarly, we isolated one BLM-deficient clone generated from TSCE5 cells transfected with the ZFN plasmids. Figure S1B shows a portion of the DNA sequence for exon 14 of the *BLM* gene in the *BLM*-disrupted TSCE5 and TSCER2 cell clones. Clone 5 derived from TSCE5 cells contained 7- and 5-bp deletions in each *BLM* allele. The deletions occurred in a region encoding the helicase domain designed to be cleaved by ZFN. Clone 2 derived from TSCER2 cells contained a 74-bp deletion and a 7-bp insertion in one allele and an 18-bp deletion in the other. Clone 10 contained 75- and 183-bp deletions in each allele. These deletions and rearrangements were likely caused by NHEJ during the repair of ZFN-induced DSBs, and they completely disrupted BLM function. Western blotting revealed no expression of the BLM helicase in BLM-TSCE5 and BLM-TSCER2 cells (see Fig. S2A).

### Characteristics of BLM-deficient cells.

**FIG 2:**
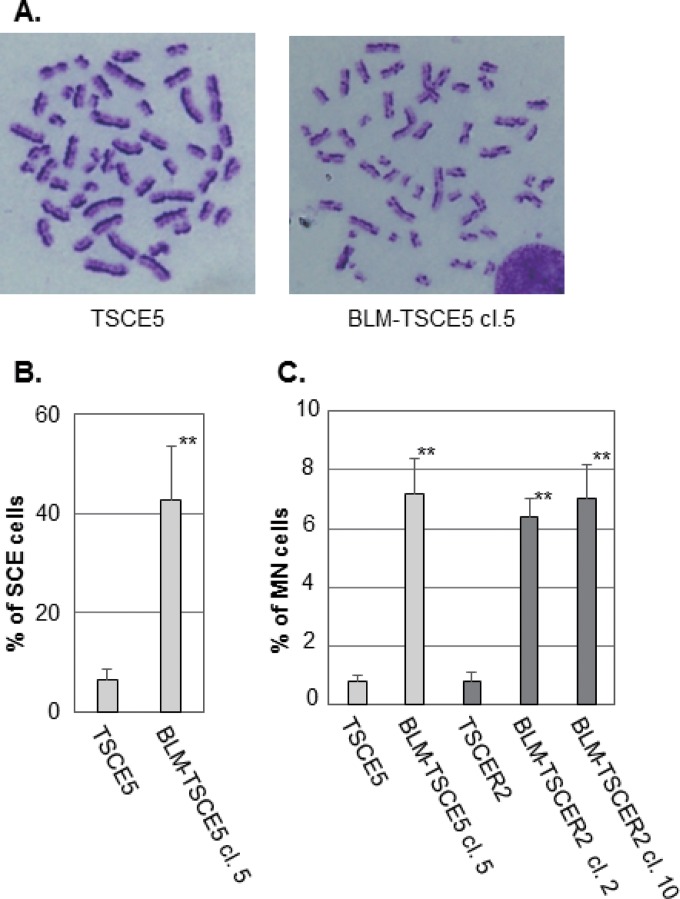
(A) Representative images of metaphase spreads from TSCE5 and BLM-TSCE5 cells used in the sister chromatid exchange (SCE) analysis. (B) Frequencies of spontaneous SCE in TSCE5 and BLM-TSCE5 cells. (C) Frequencies of spontaneous micronucleus formation in TSCE5 and TSCER2 cells and their BLM-deficient clones. The data are presented as means and standard deviations (SD) for three independent experiments. **, *P* < 0.01.

We characterized the BLM-deficient clones from TSCE5 and TSCER2 cells. The BLM-deficient cells grew slightly slower than the parent cells (see Fig. S2B in the supplemental material). A hallmark of BS cells is genomic instability that manifests as a high frequency of SCE (23). BLM-TSCE5 cells exhibited an approximately 7-fold higher frequency of SCE than that of the parent TSCE5 cells (Fig. 2A and B). We did not examine SCE in TSCER2 cells and their derivatives because TSCER2 cells are TK enzyme deficient and lose the ability to incorporate 5-bromodeoxyuridine, which is required for SCE analysis. The formation of MN is prevalent in BS cells and is closely associated with SCE (24, 33, 37). The frequencies of spontaneously generated MN for BLM-TSCE5 and BLM-TSCER2 cells were approximately 7-fold higher than those for the parent wild-type cells (Fig. 2C). These results clearly indicate that the *BLM*-deficient cell clones exhibited characteristics representative of BS cells. We used BLM-TSCE5 clone 5 and BLM-TSCER2 clone 10 for subsequent studies.

### Spontaneous thymidine kinase mutant and revertant frequencies in BLM-deficient cells.

**FIG 3:**
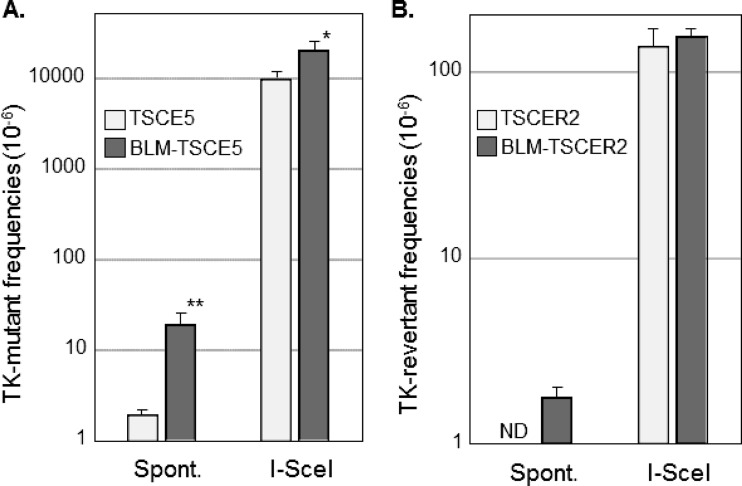
(A) Spontaneous (Spont.) and double-strand break (DSB)-induced thymidine kinase (TK) mutant frequencies in TSCE5 and BLM-TSCE5 cells. (B) Spontaneous and DSB-induced TK revertant frequencies in TSCER2 and BLM-TSCER2 cells. Cells were transfected with the I-SceI expression vector (pCBASce) to induce DSBs. The data are presented as means and SD for four independent experiments. *, *P* < 0.05; **, *P* < 0.01; ND, not detected.

TK-proficient TSCE5 cells are *TK* heterozygous and can generate TK-deficient mutants by mutation of the functional *TK* gene or by HR between alleles, which leads to LOH (Fig. 1B). Conversely, TK-deficient TSCER2 cells are *TK* compound heterozygous and can generate TK-proficient revertants only by HR between *TK* alleles (Fig. 1B). We investigated the spontaneous MF and RF of BLM-TSCE5 and BLM-TSCER2 cells, respectively. The spontaneous MF of wild-type TSCE5 cells was approximately 2 × 10^−6^, whereas BLM-TSCE5 cells exhibited an approximately 10-fold increase in MF (Fig. 3A). Spontaneous revertants from TSCER2 cells were rarely observed in BLM-proficient TSCER2 cells, and no spontaneous revertants were isolated in this study or in a previous study (15). However, revertants were spontaneously generated from BLM-TSCER2 cells at a frequency of 1.8 × 10^−6^ (Fig. 3B). These results indicate that BLM-deficient cells exhibit a mutator phenotype and likely produce mutants and revertants by interallelic HR and/or other mechanisms.

### Characteristics of spontaneously generated thymidine kinase mutants and revertants from BLM-deficient cells.

**FIG 4:**
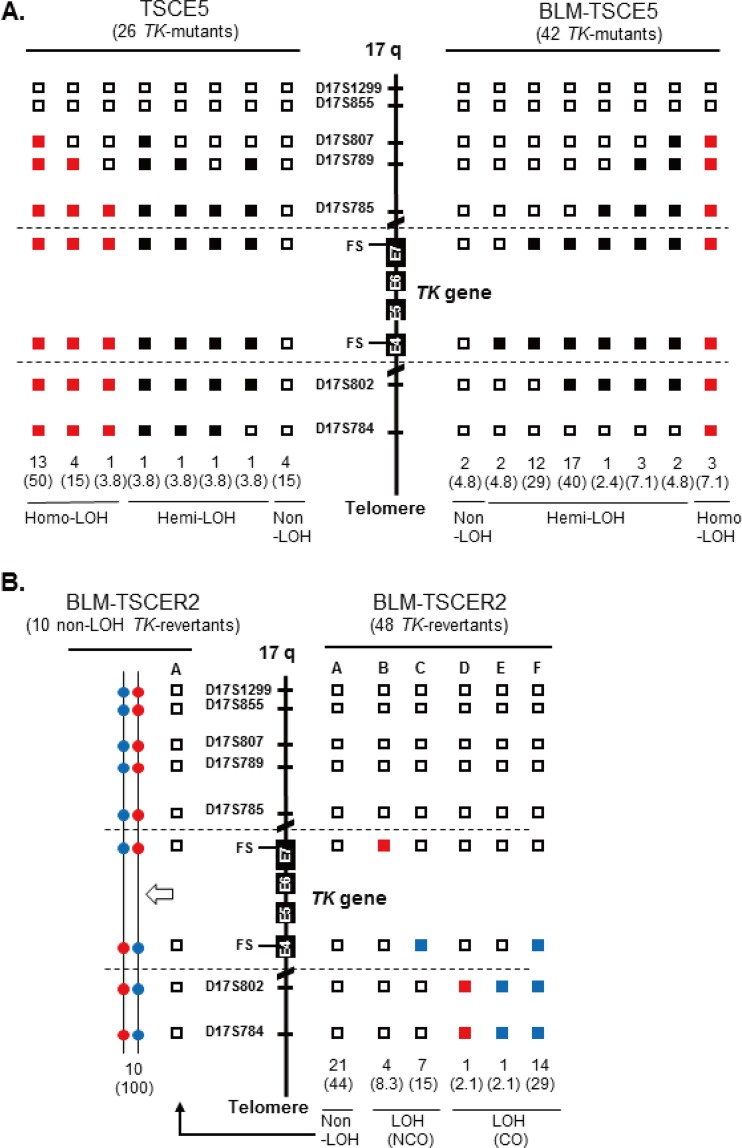
LOH analysis of chromosome 17q in spontaneous thymidine kinase (TK) mutants and TK revertants from TSCE5 and TSCER2 cells, respectively. We analyzed frameshift mutations in exons 4 and 7 of the *TK* gene and seven microsatellite loci that are heteromorphic in these cells. (A) We examined 26 and 42 TK mutants from TSCE5 and BLM-TSCE5 cells, respectively. Open squares indicate heterozygotes (non-LOH). Black and red squares indicate hemizygous LOH (hemi-LOH) and homozygous LOH (homo-LOH; a/a), respectively. (B) Forty-eight TK revertants from BLM-TSCER2 cells were examined by LOH analysis (right). Open squares indicate non-LOH. Solid red and blue squares indicate homo-LOH retaining an “a” allele and homo-LOH retaining a “b” allele, respectively. The non-LOH revertants retained heterozygosity of all DNA markers (type A). The LOH revertants were classified as the crossover (CO) type (types D to F) or the noncrossover type (types B and C). Allelotype analysis was performed for 10 of 21 non-LOH revertants (see Materials and Methods). All of the analyzed revertants resulted from HR-CO and thus retained heterozygosity.

We characterized spontaneously arising mutations and HR in BLM-deficient cells. LOH analysis was used to determine the extent of deletion or HR in chromosome 17. Quantitative analysis of PCR products was used to distinguish between deletion and HR mechanisms, which generate hemi-LOH and homo-LOH, respectively (Fig. 1B, panel 1). We previously reported the mutation spectrum for spontaneous TK mutants derived from TK6 cells (14). The mutation spectrum for TSCE5 cells was similar to that for TK6 cells: non-LOH mutations comprised 15% of mutations, hemi-LOH mutations comprised 15%, and homo-LOH mutations comprised 69% (Fig. 4A). Non-LOH mutants were likely generated by point mutations or small intragenic deletions. Hemi-LOH mutations were caused by large deletions encompassing the *TK* gene, thus resulting in large interstitial deletions or terminal deletions. Homo-LOH mutations were caused by interallelic HR. Two types of homo-LOH are possible: either the entire chromosomal arm distal to the point of crossover is homozygous (HR-CO) or the limited *TK* region is homozygous following gene conversion with a noncrossover event (HR-NCO) (Fig. 1B, panel 1). All of the homo-LOH mutations were of the CO type in TSCE5 and BLM-TSCE5 cells. The majority of TK mutants generated from TSCE5 cells were homo-LOH mutants (69%), whereas BLM-TSCE5 cells predominantly generated hemi-LOH mutants (88%). Interestingly, most of these mutants were generated by large intragenic deletions beyond the entire *TK* gene, which was never observed in TSCE5 cells. No terminal deletions were observed in BLM-TSCE5 mutants. These results indicate that TK mutants are produced mainly by HR in TSCE5 cells, whereas interstitial deletions are the main mechanism by which TK mutants are produced in BLM-TSCE5 cells (Fig. 4A).

**FIG 5:**
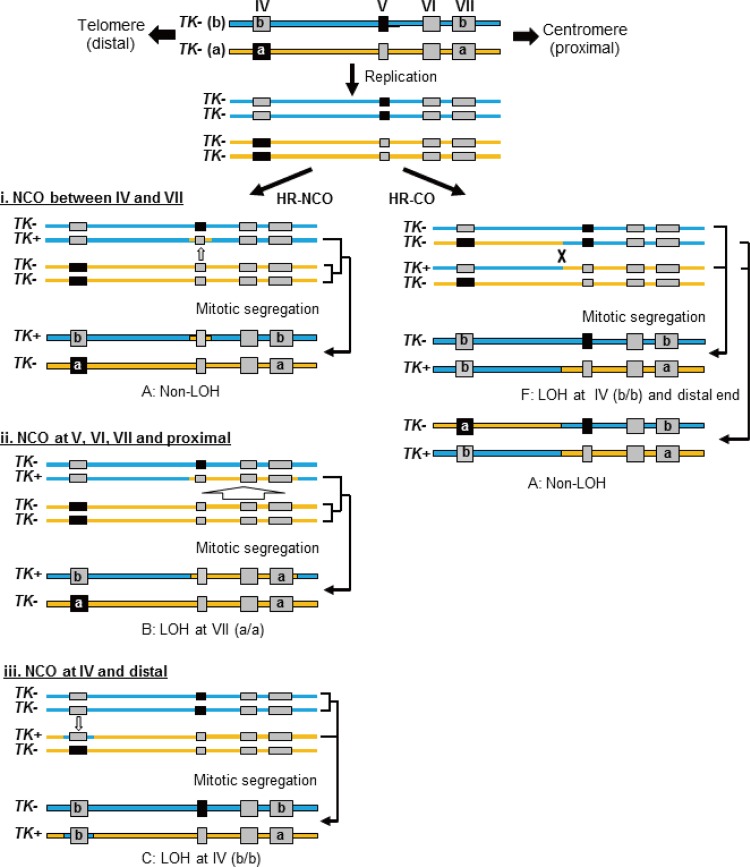
Recoverable thymidine kinase (TK) revertants created by homologous recombination (HR) from TSCER2 cells. (Left) HR without crossover (noncrossover [NCO]) can produce revertants with or without a loss of heterozygosity (LOH) at exons 4 and 7 (types A to C). When NCO is limited to exon 5, the revertants are non-LOH revertants (type A). When NCO occurs at exon 4 or 7, the revertants show LOH (types B and C). (Right) HR with CO and subsequent mitotic segregation can produce LOH (type F) or non-LOH (type A) revertants. Interallelic crossover (CO) between exons 4 and 5 and the cosegregation of recombinant and nonrecombinant chromatids into the same daughter cell produce homo-LOH from the CO point to the telomere (type F). CO between allelic chromatids and cosegregation of two recombinant chromatids into the same daughter cell generate a non-LOH event because the allelic chromosomes reciprocally exchange alleles at the CO point (type A) (right panel).

We also characterized spontaneous revertants from BLM-TSCER2 cells (Fig. 4B, right panel). Three types of revertants were identified by LOH analysis (Fig. 1B, panel 2). The non-LOH revertants maintained heterozygosity for all polymorphic loci and represented the majority of events (44%) (Fig. 4B, type A). The second type of revertant had a homo-LOH limited to the *TK* gene (23.3%) that was generated by interallelic HR-NCO (Fig. 4B, types B and C). LOH was observed at markers on exon 7 (a/a) or exon 4 (b/b). The third type of revertant had a homo-LOH extending to the distal end of the chromosome (33.2%) that was caused by interallelic HR-CO (Fig. 4B, types D to F). Non-LOH revertants (type A) are theoretically generated by one of two HR mechanisms. One mechanism consists of a short-tract gene conversion, caused by HR-NCO, which is limited to exon 5 and does not extend to exon 4 or 7. The second mechanism consists of HR-CO between exons 4 and 5 following the segregation of two recombinant sister chromatids into the same daughter cell (Fig. 5). The reciprocal interallelic exchanges during HR-CO can generate revertants that maintain heterozygosity. To distinguish between CO and NCO revertants, we conducted an allelotype analysis of 10 non-LOH revertants spontaneously generated from BLM-TSCER2 cells (see Fig. S3 in the supplemental material). This method enabled us to discriminate the lineage of the alleles. All revertants were generated by interallelic HR-CO, and no revertant was produced by HR-NCO (Fig. 4B, left panel). Theoretically, the mitotic segregation after HR-CO must equally produce LOH and non-LOH revertants (Fig. 5). In our study, 44% of revertants showed non-LOH mutations (type A) and 29% of revertants showed LOH-CO mutations (type F). Two revertants maintained heterozygosity at the markers on exons 4 and 7 and exhibited LOH until the distal end of the chromosome (types D and E). These revertants were presumably generated by a second crossover between the *TK* and 17S802 loci on chromosome 17q. These results indicate that the majority of spontaneous revertants from BLM-TSCER2 cells were caused by interallelic HR-CO.

### DSB-induced thymidine kinase mutant and revertant frequencies in BLM-deficient cells.

TSCE5 and TSCER2 cells have a homing endonuclease I-SceI site in intron 4 of the *TK* gene (Fig. 1A). The expression of the I-SceI enzyme generates a DSB in the *TK* gene in TSCE5 and TSCER2 cells and their BLM-deficient counterparts. The DSB-induced *TK* MF for TSCE5 cells was 0.95 × 10^−2^, which is 5,000-fold higher than the spontaneously induced MF (Fig. 3A). For BLM-TSCE5 cells, the DSB-induced *TK* MF was 2.9 × 10^−2^, which is 2.1-fold higher than that for TSCE5 cells. However, BLM-TSCE5 cells exhibited a 10-fold higher spontaneous MF than that of TSCE5 cells (Fig. 3A). The DSB-induced *TK* RF for TSCER2 cells was 1.4 × 10^−4^, whereas no spontaneous revertants were observed in TSCER2 cells (Fig. 3B). The DSB-induced *TK* RF for BLM-TSCER2 cells was 1.6 × 10^−4^, which is 87-fold higher than the spontaneous RF but not significantly different from the RF for wild-type TSCER2 cells. These results indicate that a DSB strongly induced TK mutants and TK revertants in wild-type and BLM-deficient cells; however, the spontaneously generated mutator phenotype was not exacerbated by DSBs.

### Characteristics of DSB-induced mutations in BLM-deficient cells.

We characterized DSB-induced TK mutants derived from TSCE5 and BLM-TSCE5 cells. Mutants with small deletions caused by NHEJ and short-tract gene conversions caused by HR-NCO that do not affect TK function are not recovered as TFT-resistant mutants in the TSCE5 assay because the I-SceI site is inserted into intron 4 of the functional *TK* allele, 75 bp upstream of exon 5 (Fig. 1A). Thus, recovery of TK mutants by drug selection may be biased. To avoid selection bias and investigate small genetic changes induced by DSBs, we randomly isolated 480 clones each, without TFT selection, from wild-type TSCE5 and BLM-TSCE5 cells after I-SceI expression and directly analyzed their DNAs. The high *TK* MF (>10^−2^) caused by site-specific DSBs enabled the analysis. Table 1 shows the spectra of DSB-induced mutations in TSCE5 and BLM-TSCE5 cells. We identified 15 mutants from the 480 non-drug-selected TSCE5 cells and 37 mutants from the 480 non-drug-selected BLM-TSCE5 cells. Their MFs were 3.1% and 7.7%, respectively. These ratios corresponded well with the *TK* MF obtained by TFT selection of TSCE5 and BLM-TSCE5 cells (2.1-fold) (Fig. 3A). Seventy-three percent of mutants derived from TSCE5 cells had small or medium-sized deletions of less than 1 kb, and the remaining 27% mutants had complicated DNA rearrangements (see Fig. S4A in the supplemental material). This result is consistent with our previous reports (15, 17). The mutation spectrum for BLM-TSCE5 mutants was similar to that for TSCE5 cells. In addition, large deletions of more than 1 kb were prevalent. Single-base insertions, interallelic recombination, and translocation were observed less frequently (see Fig. S4B for details). Single-base insertions and interallelic recombination were previously observed in wild-type TSCE5 cells (17); therefore, these genetic changes do not specifically occur in BLM-deficient cells. Our results revealed that NHEJ works appropriately in BLM-TSCE5 cells and that the DSB resulted mainly in deletions and rearrangements. However, gross structural changes were observed more frequently in BLM-TSCE5 cells than in wild-type TSCE5 cells.

**TABLE 1:** Mutation analysis of nonselected TSCE5 and BLM-TSCE5 mutants after I-SceI expression

Mutation type	No. (%) of mutants^*a*^	
	TSCE5 cells (480 clones)	BLM-TSCE5 cells (480 clones)
Small deletion (<60 bp)	8 (53)	18 (49)
Medium-sized deletion (60 bp–1 kb)	3 (20)	3 (8.1)
Large deletion (>1 kb)	0	6 (16)
Insertion (1 bp)	0	2 (5.4)
Rearrangement	4 (27)	5 (14)
Recombination	0	2 (5.4)
Translocation	0	1 (2.7)
Total	15 (3.1)	37 (7.7)

a Except for the last row. The data in the last row are numbers (%) among all clones.

### Characteristics of DSB-induced thymidine kinase revertants generated by homologous recombination in BLM-deficient cells.

**FIG 6:**
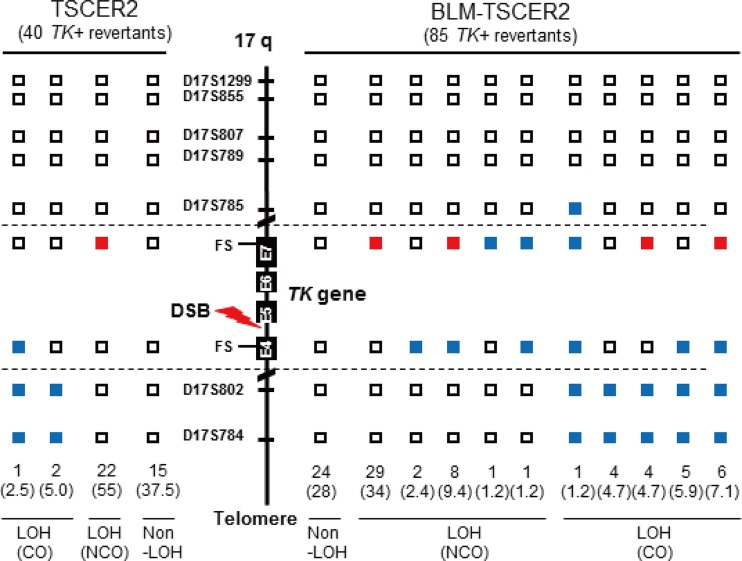
LOH analysis of chromosome 17q with double-strand break-induced thymidine kinase (TK) revertants from TSCER2 and BLM-TSCER2 cells. We analyzed frameshift mutations in exons 4 and 7 of the *TK* gene and seven microsatellite loci that are heteromorphic in these cells. We examined 40 TK revertants from TSCER2 cells (left) and 85 TK revertants from BLM-TSCER2 cells (right). Open squares indicate non-LOH. Solid red and blue squares indicate homo-LOH retaining an “a” allele and homo-LOH retaining a “b” allele, respectively. The LOH revertants were classified into crossover and noncrossover types.

**FIG 7:**
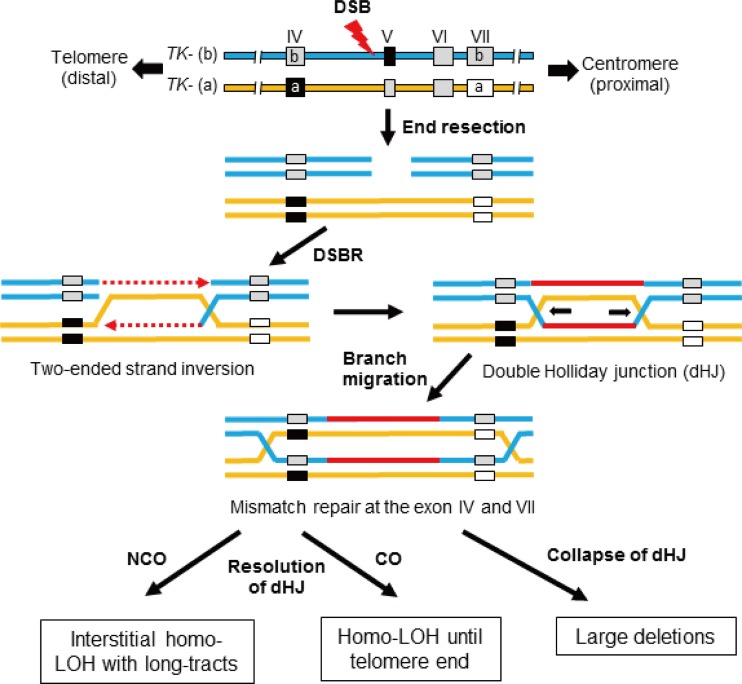
Proposed mechanism of LOH generation during homologous recombination to repair a DSB at the I-SceI site 75 bp upstream from exon 5 of the thymidine kinase gene (*TK*). According to the DSB repair model, the DSB forms an intermediate double Holliday junction (dHJ) after end resection and strand inversion. Branch migration of the double HJ leads to a long heteroduplex DNA, and thus mismatches at exons 4 and 7 are created. The mismatches are repaired, resulting in a region of localized LOH. After resolution of the double HJ following the segregation of sister chromatids, either crossover or noncrossover LOH occurs. BLM helicase suppresses the elongation of branch migration and crossover of the double HJ intermediates during HR repair. BLM helicase deficiency causes collapse, abnormal elongation, and/or preferable resolution to crossover of double HJs, resulting in large-scale LOH and deletions.

We also characterized DSB-induced TK revertants from TSCER2 and BLM-TSCER2 cells. These revertants were classified into three types: non-LOH, LOH-NCO, and LOH-CO. Among the 40 revertants derived from TSCER2 cells, 15 were non-LOH revertants (37.5%), 22 were LOH-NCO revertants (55%), and 3 were LOH-CO revertants (7.5%) (Fig. 6). Conversely, among the 85 revertants derived from BLM-TSCER2 cells, 28% were non-LOH revertants, 49% were LOH-NCO revertants, and 24% were LOH-CO revertants. We did not perform an allelotype analysis of the non-LOH revertants; thus, it is unclear if they were caused by CO or NCO. However, LOH-CO occurred more frequently in BLM-TSCER2 cells than in wild-type TSCER2 cells (24% versus 7.5%), indicating that CO is the predominant mechanism when a DSB is repaired by HR in BLM-deficient cells (Fig. 5). Several LOH patterns were observed within the *TK* gene, regardless of CO or NCO. Possible mechanisms by which LOH is produced are illustrated in Fig. 7. The DSB induces a double HJ intermediate during HR repair. Branch migration of this double HJ leads to a long heteroduplex DNA, which creates mismatches at exons 4 and 7. These mismatches are repaired, resulting in a region of localized LOH. Wild-type TSCER2 cells showed LOH limited to exon 4 or 7, whereas BLM-TSCER2 cells produced LOH at both exons 4 and 7, indicating that branch migration proceeded further in BLM-deficient cells than in wild-type cells. For example, a BLM revertant (Fig. 6) showed LOH at the D17S785 locus, which is 200 kb away from the DSB.

## DISCUSSION

### The high frequency of spontaneously occurring interallelic HR is a representative mutator phenotype of BLM helicase-deficient human cells.

We determined the mutator phenotype of BLM-deficient human cells. More specifically, BLM-TSCER2 cells produced spontaneously arising TK revertants through interallelic HR, which was never observed in wild-type TSCER2 cells (Fig. 3B). Crossover events occur predominantly during interallelic HR, resulting in LOH or in reciprocal exchanges of alleles from the crossover points to the telomere end. Two TK revertants (types D and E in Fig. 4B) were generated by double crossover: the first between exons 4 and 5 of the *TK* gene and the second between the *TK* gene and the 17S802 locus, located approximately 2 Mb from the distal end of the *TK* locus (Fig. 1A). These findings clearly suggest that the crossover events between homologous chromosomes preferably occur in BS cells. A hallmark characteristic of BS cells is a highly elevated frequency of SCE (Fig. 2A and B) (22, 23). SCE is the result of sister chromatid recombination (SCR) with crossover and can be assayed cytogenetically. Noncrossover SCR events must occur frequently to repair spontaneously arising DSBs during DNA replication. However, these events cannot be detected cytogenetically. The increases in SCE and interallelic HR with crossover are evidence of mutator phenotypes with a common mechanism.

Noncrossover revertants were observed infrequently (23.5%) in BLM-TSCER2 cells (types B and C in Fig. 4B). These revertants were generated by gene conversion involving exon 7 (type B) or exon 4 (type C). The tract lengths of the conversion remain unknown because we could not analyze the appropriate DNA markers near exon 7 or exon 4. Type B LOH revertants were generated by gene conversion at exon 5 and exon 7 (Fig. 5), and the tract length was estimated to be more than 1 kb (Fig. 1). According to the allelotype analysis, there were no noncrossover revertants exhibiting gene conversion limited to either exon 4 or 5 of the *TK* gene (Fig. 5, left panels, type A). In other words, the gene conversions occurring on exon 5 always involved exons 4 and 7, with tract lengths estimated to be more than 8 kb (Fig. 1). In mammalian cells, somatic gene conversion tracts are typically less than 1 kb long (38). Our findings indicate that if gene conversion without crossover occurs in BLM-deficient cells, the tract length is much longer than that in wild-type cells. These results reinforce the view that the increases of interallelic HR with crossover and long-tract gene conversion without crossover may be key contributors to the generation of large-scale LOHs.

### Large deletions are major spontaneous mutation events in BLM helicase-deficient human cells.

BLM-TSCE5 cells exhibited a much higher spontaneous TK mutant frequency than that of wild-type TSCE5 cells (Fig. 3A). The observations of a high frequency of interallelic HR and increased SCEs in BLM-TSCER2 cells prompted us to expect that homo-LOH through interallelic HR strongly contributes to the increase in mutants in the *TK* gene mutation assay. However, the majority of TK mutants from BLM-TSCE5 cells were hemi-LOH mutants (88%), and homo-LOH mutations accounted for 7.1% of the mutants. This result indicates that the deficiency of BLM helicase preferably induces deletions rather than homo-LOH by interallelic HR. Interestingly, most of these deletions are large intragenic deletions of more than 2 Mb that cover the entire *TK* gene. These intragenic deletions were rarely observed in wild-type TSCE5 cells (Fig. 4A). Consistently, Tachibana et al. (39) reported that BS cells display a higher spontaneous *HPRT* mutation rate than that of wild-type cells and predominantly produce large deletions that include the entire *HPRT* gene. However, these results do not mean that BLM-deficient cells lose HR activity. BLM-TSCE5 cells exhibited a 10-fold increase in TK mutations, and homo-LOH accounted for only 7.1% of these, which suggests that HR activity is maintained in BLM-deficient cells. Thus, BLM helicase deficiency does not affect HR activity but rather a change in HR, characterized by the preferential occurrence of crossover and long-tract gene conversion in BLM-TSCE5 cells. However, the reason for the high frequency of large deletions (hemi-LOH) in BLM-TSCE5 cells remains unknown.

BLM helicase forms a complex with topoisomerase IIIα (hTOPO IIIα) and hRMI1 (37, 40, 41). This complex dissolves double HJs, thus resulting in the generation of noncrossover products (see below). This function is conserved in many organisms to maintain genomic integrity (42). BLM helicase is also involved in faithful chromosome segregation (37). During mitosis, BLM helicase, hTOPO IIIα, and hRMI1 colocalize to anaphase bridges, which are abundantly generated during anaphase in normal cells, and subsequently dissolve them. BLM-deficient cells display a high frequency of anaphase bridges (37, 43). Anaphase bridges are thought to generate micronuclei as a consequence of the breakage of the bridges by mitotic spindle force (44). BS cells exhibit micronuclei at high frequencies (Fig. 2B) (24, 33). The breakdown of anaphase bridges may cause partial loss of the chromosome, thus resulting in large deletions. A failure to prevent the progression of anaphase bridges and their subsequent collapse may be an underlying mechanism for the high frequency of micronucleus formation and large deletions in BLM-TSCE5 cells. Micronuclei may contribute to subsequent genomic instability via gene dosage imbalances or chromothripsis (45, 46).

### The genetic consequences of DSBs in BLM helicase-deficient human cells and wild-type human cells are similar.

We introduced a DSB into the *TK* gene by using I-SceI and then traced the genetic consequences. The DSB strongly induced TK mutants in both wild-type TSCE5 cells and BLM-TSCE5 cells (Fig. 3A). The induced mutant frequency was approximately 2-fold higher in BLM-TSCE5 cells than in TSCE5 cells (Fig. 3A). DSBs in mammalian cells are repaired by either NHEJ or HR (3, 5, 6). NHEJ causes deletions and HR causes gene conversion, leading to hemi-LOH and homo-LOH TK mutants, respectively. Because the I-SceI site is located in intron 4 of the functional *TK* gene, 75 bp upstream of exon 5 (Fig. 1), small deletions and short-tract gene conversions that do not extend to exon 5 cannot be recovered as TK mutants in the TSCE5 system. The higher frequency of DSB-induced TK mutants in BLM-TSCE5 cells implies that large-scale deletions and/or gene conversions occur predominantly in BLM-deficient cells.

To precisely characterize the mutation spectrum, we randomly isolated 480 non-drug-resistant clones from TSCE5 and BLM-TSCE5 cells after transfection with the I-SceI vector, and we directly analyzed their DNAs. We observed 15 mutants (3.1%) from TSCE5 cells and 37 mutants (7.7%) from BLM-TSCE5 cells. Eleven mutants from TSCE5 cells (73%) showed small or medium-sized deletions of less than 1 kb. We previously conducted the same experiment to characterize the non-drug-resistant mutants from DSB-induced TSCE5 cells and demonstrated that approximately 80% of the mutants featured small deletions (17). Thus, the majority of DSBs in wild-type cells were repaired by NHEJ, resulting in small deletions. Conversely, large deletions were also observed at a high frequency in BLM-deficient cells (16%) (Table 1), implying that the higher mutant frequency for BLM-TSCE5 cells in the *TK* mutation assay was due to large deletions (>75 bp) involving exon 5. Other genetic changes, including insertions, rearrangements, recombinations, and translocations, were observed infrequently in BLM-deficient cells.

Yamanishi et al. (28) developed an experimental system using mouse embryonic stem (ES) cells to detect LOH mutations in the adenine phosphoribosyltransferase gene. In this system, BLM expression and DSBs caused by I-SceI can be controlled, and the authors demonstrated that BLM-suppressed ES cells predominantly exhibited deletions after DSB repair. Complicated rearrangements that included deletions and insertions, whose breakpoints were clustered in inverted repeats and joined with microhomology, were also observed in BLM-suppressed cells. Thus, Yamanishi et al. concluded that a loss of BLM helicase function enhanced microhomology-mediated genomic rearrangements and led to increased LOH events. However, we observed these complicated rearrangements not only in BLM-TSCE5 cells but also in wild-type TSCE5 cells (Table 1). A DNA sequence analysis revealed that larger deletions were associated with complicated rearrangements in BLM-TSCE5 cells (see Fig. S4B in the supplemental material), but microhomology sequences at the junction of the inverted repeat were found in both wild-type and BLM-deficient mutants (15). These complicated rearrangements do not contribute significantly to the mutator phenotype of BLM-TSCE5 cells. Our results indicate that DSBs are repaired predominantly by NHEJ, resulting in deletions in both wild-type and BLM-deficient cells. Although BLM-deficient cells develop a tendency to produce large deletions, the mechanism and efficiency of NHEJ repair are maintained. Because the frequencies of large deletions arising spontaneously and induced by the DSB are absolutely different (Fig. 3), the specific mechanisms related to spontaneously arising deletions in BLM-TSCE5 cells are not significantly manifested under the conditions for repairing DSBs.

### DSBs repaired by HR result in large-scale LOH in BLM helicase-deficient human cells.

We introduced a DSB by expressing I-SceI in the *TK* gene of TSCER2 cells and investigated the HR mechanism for DSB repair. The revertant frequencies after introduction of a DSB did not differ between wild-type and BLM-TSCER2 cells (Fig. 3B), indicating that the quantitative activity of HR for DSB repair is maintained in BLM-deficient cells and that HR does not compete with NHEJ for DSB repair. Two models have been proposed for DSB repair by HR: the synthesis-dependent strand-annealing (SDSA) model and the DSB repair (DSBR) model (Fig. 7) (47, 48). In the DSBR model, a double HJ is formed, which results in crossover and noncrossover revertants. In contrast, the SDSA model always generates noncrossover revertants. When HR is used to repair DSBs, repair is achieved predominantly by the SDSA model (49). However, these models are thought to occur between sister chromatids after DNA replication, and it is unclear whether the SDSA and DSBR models function between homologous alleles. Regardless, the TSCER2 system is unable to detect most SDSA-mediated revertants because the DNA tract of strand invasion in the SDSA model is short and the DNA repair is completed before strand invasion and DNA synthesis reach the site of *TK* mutation at exon 5, which is 75 bp away from the I-SceI site (Fig. 1A). Thus, most revertants in the TSCER2 system were likely generated by the DSBR mechanism.

BLM helicase forms a complex with hTOPO IIIα, which can break and rejoin DNA to alter its topology. Wu and Hickson (40) reported that BLM helicase and hTOPO IIIα affect the resolution of double HJs via a mechanism of “double-junction dissolution,” which is distinct from classical HJ resolution and prevents crossover. Deficiency of such an activity may explain the BS cell phenotype, including the high frequencies of SCE and LOH by crossover. The RecQ helicase in budding yeast, Sgs1, has a similar role during interchromosomal recombination (50, 51). Sgs1 suppresses crossover by reverse branch migration of double HJs until they are resolved by noncrossover events. In the absence of Sgs1, double HJs are free to engage in forward branch migration, elongating heteroduplex DNA and increasing the gene conversion tract length, which is consequently resolved, leading to crossover (51).

The results of our TSCER2 studies firmly support this concept. The high frequency of LOH with crossover and the increase in long-tract gene conversion after the resolution of double HJs are evident in BLM-TSCER2 cells. A DSB occurring at the I-SceI site in intron 4 of the *TK* gene forms a double HJ (Fig. 7). Reciprocal branch migration and DNA synthesis elongate heteroduplex DNA and produce mismatches at exons 4 and 7 that contain frameshift mutations. The mismatches are repaired, resulting in LOH at exons 4 and 7 depending on the segregation of the sister chromatids. Because the mutation at exon 4 is critical for generating revertants, the *TK* revertants must be +/+; b/b or +/−; b/a. However, any pattern is possible at exon 7, because the mutation at exon 7 is not associated with the phenotype. In wild-type TSCER2 revertants, LOH is limited to exon 4 or 7, whereas some BLM-deficient TSCER2 revertants showed LOH at both exons 4 and 7, regardless of crossover or noncrossover. The length of the heteroduplex DNA was at least 8 kb (Fig. 1A).

Our LOH analysis revealed that 7.5% of LOH revertants from wild-type TSCER2 cells resulted from crossover, whereas BLM-TSCER2 cells produced crossover revertants at a frequency of 24% (Fig. 7). This result indicates that crossover events occur preferably in BLM-deficient cells. However, LOH by noncrossover events remained the predominant event in BLM-TSCER2 cells (48%), implying that BLM helicase is not the only factor to determine the occurrence of crossover or noncrossover events during the resolution of a double HJ. One of the BLM-TSCER2 revertants showed LOH at the D17S785 locus, which is 200 kb away from the *TK* gene (i.e., the branch migration extended more than 200 kb in BLM-deficient cells). This is the first report to estimate the length of heteroduplex DNA created by branch migration in a double HJ in a mammalian genome and to demonstrate that the length is much longer in BLM-deficient cells than in wild-type cells. The long heteroduplex DNA with double HJs may be unstable. Its collapse or abnormal resolution may cause large-scale genomic alterations in the genome. The large deletions, complex DNA rearrangements, and translocations observed in BLM-TSCE5 cells (see Fig. S4B in the supplemental material) may be generated by the collapse of the long heteroduplex DNA (28).

In summary, BLM-deficient human cells exhibit a high spontaneous frequency of interallelic HR with crossover; furthermore, noncrossover events occur at a low frequency, but their tract lengths are much longer than those in wild-type cells. Despite the highly interallelic HR events, BLM-deficient cells predominantly produce hemi-LOH by spontaneous deletion. These mutator phenotypes manifest as a method of DSB repair. Similarly to those in wild-type cells, both NHEJ and HR function appropriately in BLM-deficient cells, resulting in hemi-LOH and homo-LOH, respectively. However, the scale of the LOH is extended in BLM-deficient cells and features large deletions and long-tract gene conversions with crossover. BLM helicase suppresses the elongation of branch migration and crossover of double HJ intermediates during HR repair to maintain genomic stability, and its deficiency causes collapse, abnormal elongation, and/or preferable resolution to crossover of double HJs, resulting in large-scale LOHs (deletions or homozygous alleles), SCEs, and MNs. Large-scale LOHs increase the possibility of homozygous mutations in tumor suppressor genes. SCEs and the formation of MNs may cause subsequent genomic instability, leading to DNA rearrangements and translocations. Thus, these features likely comprise the underlying mechanism of cancer predisposition in BS.

## Supplementary Material

Supplemental material
