# Supplementary material for: Mutator Phenotype and DNA Double-Strand Break Repair in BLM Helicase-Deficient Human Cells
Source: Mol Cell Biol. 2016 Nov 14;36(23):2877–89. doi: 10.1128/MCB.00443-16 (PMC5108877; doi:10.1128/MCB.00443-16)
Supplement: Supplemental material [file supp_36_23_2877__index.html]

Mutator Phenotype and DNA Double-Strand Break Repair in BLM Helicase-Deficient Human Cells — Supplemental material 

# Mutator Phenotype and DNA Double-Strand Break Repair in BLM Helicase-Deficient Human Cells

## Supplemental material

- Supplemental file 1 -

  Legends to Fig. S1 to S4

  PDF, 87K
- Supplemental file 2 -

  Fig. S1 (Target sequence of part of exon 14 of human *BLM*), S2 (Expression of *BLM* genes), S3 (Generation of LOH revertants by HR with or without crossover), and S4 (Sequence analysis of nonselected TSCE5 mutants)

  PDF, 135K
