# Supplementary material for: Mutator Phenotype and DNA Double-Strand Break Repair in BLM Helicase-Deficient Human Cells
Source: Mol Cell Biol. 2016 Nov 14;36(23):2877–89. doi: 10.1128/MCB.00443-16 (PMC5108877; doi:10.1128/MCB.00443-16)
Supplement: Supplemental material [file MCB.00443-16_zmb999101353so1.pdf]

LEGENDS OF SUPPLEMENTAL MATERIALS

Supplement 1. A) The target sequence of part of Exon 14 of the human *BLM* gene is illustrated. The sequences recognized by zinc-finger nuclease (ZFN) 1 and ZFN2 are in the coding sequence of the helicase domain of the *BLM* gene. B) Sequence analyses of *BLM*-deficient clones from TSCE5 and TSCER2 cells. Each clone had two dysfunctional *BLM* alleles by deletion (Δ) or rearrangement (Δ + Ins). Microhomologous sequences at junctions are shown in light blue.

Supplement 2. A) Western blotting analysis of the expression of *BLM* genes in *BLM*-deficient clones and their parent cells. β-tubulin was used as a loading control. B) Population doublings of TSCE5 and TSCER2 cells and their *BLM*-deficient clones. The data are presented as the mean ± standard deviation (SD) of three independent experiments; bars indicate SD. $^{*}P < 0.05$.

Supplement 3. Non-loss of heterozygosity (LOH)-revertants from TSCER2 cells are generated by homologous recombination (HR) with crossover (CO) or HR without crossover (NCO). They can be distinguished by allelotype analysis (see Materials and methods). We isolated some spontaneous *thymidine kinase* (*TK*) mutants from revertants generated through HR-CO and analyzed the LOH patterns at the polymorphic locus (17S802; Fig. 1) on the distal end of the *TK* gene. If the mutant was a/a, the original revertant was generated by HR-NCO. If it was b/b, the original revertant was created by HR-CO.

Supplement 4. Detailed sequence analysis of non-selected TSCE5 mutants (A) and BLM-TSCE5 mutants (B) after I-*Sce*I expression (Table 1). Underlining indicates the I-*Sce*I recognition sequences. Red represents deleted sequences, and green represents

inserted sequences. Four of six large deletion mutants from BLM-TSCE5 were not examined by sequence analysis because the polymerase chain reaction products could not be obtained. Loss-of-heterozygosity analysis for two recombination mutants (BLM_139 and BLM_240) from BLM-TSCE5. The I-*Sce*I sequence was lost and replaced by the sequence from Intron 4 of the other allele (not deleted) in both mutants. BLM_139 showed homo-LOH in Exons 4 and 7 (a/a) of the *thymidine kinase* (*TK*) gene. BLM_240 showed homo-LOH in Exon 7 (a/a) of the *TK* gene. Spectrum karyotype analysis of a mutant cell with translocation (BLM_89) and parental BLM-TSCE5 cells. The big arrow points to t(2, 17), in which a part of Chromosome 17 has unreciprocally translocated to Chromosome 2.
