# Supplementary material for: Mutator Phenotype and DNA Double-Strand Break Repair in BLM Helicase-Deficient Human Cells
Source: Mol Cell Biol. 2016 Nov 14;36(23):2877–89. doi: 10.1128/MCB.00443-16 (PMC5108877; doi:10.1128/MCB.00443-16)
Supplement: Supplemental material [file MCB.00443-16_zmb999101353so2.pdf]

A.

52081 tccaactagt ggggacatga ttttcgtcaa gattacaaaa

52121 gaatgaatat gcttcgccag aagtttcctt ctgttccggt

FokI ZFN

52161 gatggctctt acggccacag ctaatcccag ggtacagaag

ZFN FokI

52201 gacatcctga ctcagctgaa gattctcaga cctcaggtgt

52241 aagttgttgc acgtcacgta

B.

BLM-TSCE5

clone 5
52171 acg(Δ7 bp)ctaatcccag 52190
52171 acggccaca(Δ5 bp)tcccag 52190

BLM-TSCER2

clone 2
52151 c(Δ74 bp + Ins 7 bp)caga 52230
52161 gatggctc(Δ18 bp)ccag 52190

clone 10
52171 acggc(Δ75 bp)acgtcacgta 52260
52171 acggcc(Δ183 bp)caagttgtaac 52370

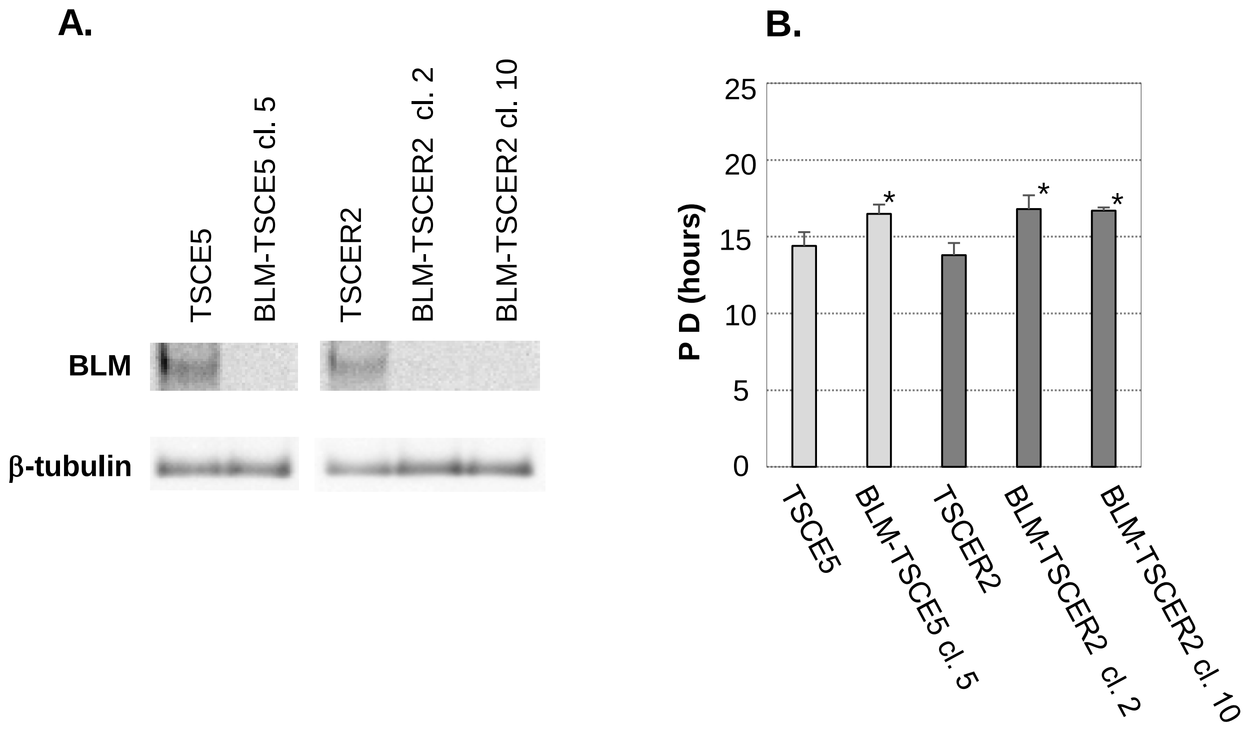

Supplement 2

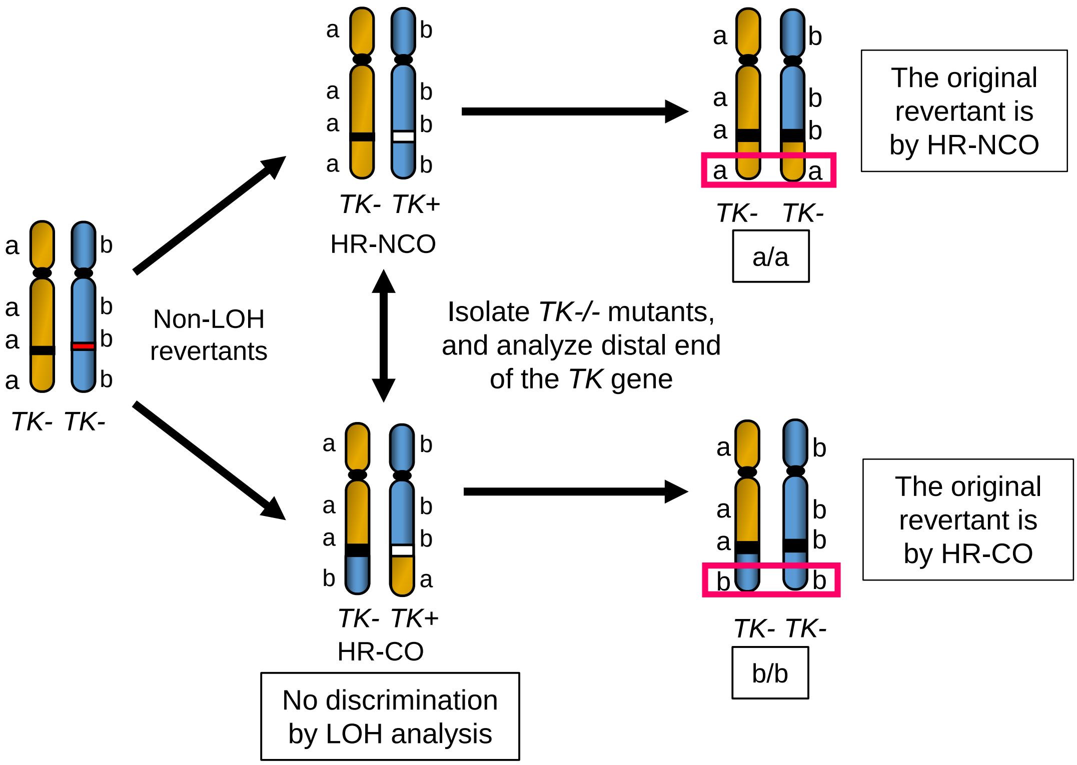

a a a a
b b b b
TK- TK-
Non-LOH revertants
a a a a
b b b b
TK- TK+
HR-NCO
Isolate TK-/- mutants, and analyze distal end of the TK gene
a a a b
b b b a
TK- TK+
HR-CO
No discrimination by LOH analysis
a a a a
b b b a
TK- TK-
a/a
The original revertant is by HR-NCO
a a a b
b b b b
TK- TK-
b/b
The original revertant is by HR-CO

A. Non-selected mutants from TSCE5

1) Small deletion (< 60 bp); 8 clones

TSCE_141 agatccattaccctgttatccctactctcgaggatct (Δ14)

TSCE_213 agatccattaccctgttatccctactctcgaggatct (Δ2)

TSCE_225 gggccaaatggccggagttgtcagatccattaccctgttatccctactctcg (Δ30)

TSCE_302 agatccattaccctgttatccctactctcgaggatct (Δ4)

TSCE_396 agatccattaccctgttatccctactctcgaggatct (Δ1)

TSCE_431 tgtcagatccattaccctgttatccctactctcgaggatctggcagcgt (Δ31)

TSCE_434 agatccattaccctgttatccctactctcgaggatct (Δ9)

TSCE_311 ttactccgggccaaatggccggagttgtcagatccattaccctgttatccctactctcgagg (Δ50)

2) Middle deletion (60 bp – 1kb); 3 clones

TSCE_262
ttgccaaggtcacacagataatgggtccagcgaagagtggtgtccgagcccaaggcagcaggcctttggccactgcagtgttaaacagcaca
gctggtgtggaagtccggtgctgagtcctgggtacctggactcggagggaagctggctgcaggggggaagggggctgcgcagttgtggatgtacct
gtcgtctgctgggggggcgtgcgggtggacacagtcccccggcctggggagcctcgtgggagaattaagagttactccgggccaaatggccgg
agttgtcagatccattaccctgttatccctactctcgagg (Δ295)

TSCE_306
gggccaaatggccggagttgtcagatccattaccctgttatccctactctcgaggatctggcagcgtcttcgctggggctccagggagctgctgctg
gggtggaagctctcacactctttctccacgtgccctttccagttccctgacatcgtggagttctgcgagg (Δ144)

TSCE_392
cggcctggggagcctcgtgggagaattaagagttactccgggccaaatggccggagttgtcagatccattaccctgttatccctactctcgaggat
ctggcagcgtcttcgctggggctccagggagctgctgctggggtggaagctct (Δ129)

3) Rearrangements; 4 clones

TSCE_9 agatccattaccctgttatccctgcgaagactctcgaggatct (Δ9 / +6 unknown)

TSCE_393 ctccgggccaaatggccggagttgtcagatccattaccctgttatccctactctcgaggatctggcag (Δ23 / Δ9)

TSCE_283
ggagttgtcagatccattaccctgttatccctactctcgaggatctggcagcgtcttcgctggggctccagggagctgctgctggggtggaagctctc
acactctttctccacgtgccctttccagttccctgacatcgtggagttctgcgaggccatggccaacgccgggaagaccgtaattgtggctgcactg
gatgggaccttccagaggaaggtaaggcgtctgatccaggtctggagctgggattgaggagggcaagaggcttctggatggcacagagaca
ccagctctgggtgaccagggctcagccaccacagggttacggccgagctgctcaggccttggctgagcc (Δ37 / +117 inverted insertion of part of TK gene / Δ179)

TSCE_214
ggggagcctcgtgggagaattaagagttactccgggccaaatggccggagttgtcagatccattaccctgttatccctactctcgaggatctggca
gcgtcttcgctggggctccagggagctgctgctggggtgggagctgctgctggggtggaagctctcaca (Δ22 / +18 inverted insertion of part of TK gene)

B. Non-selected mutants from BLM-TSCE5

1) Small deletion (< 60 bp); 18 clones

BLM_19 agatccattaccctgttatccctactctcgaggatct (Δ9)

BLM_47 agatccattaccctgttatccctactctcgaggatct (Δ11)

BLM_68 agatccattaccctgttatccctactctcgaggatct (Δ9)

BLM_75 agatccattaccctgttatccctactctcgaggatct (Δ16)

BLM_83 agatccattaccctgttatccctactctcgaggatct (Δ16)

BLM_145 agatccattaccctgttatccctactctcgaggatct (Δ3)

BLM_147 agatccattaccctgttatccctactctcgaggatct (Δ24)

BLM_233 agatccattaccctgttatccctactctcgaggatct (Δ9)

BLM_268 tcagatccattaccctgttatccctactctcgaggatctggcag (Δ31)

BLM_328 agatccattaccctgttatccctactctcgaggatct (Δ4)

BLM_361 agatccattaccctgttatccctactctcgaggatct (Δ1)

BLM_356 ttaccctgttatccctactctcgaggatctggcagcgtcttcgct (Δ31)

BLM_364 agatccattaccctgttatccctactctcgaggatct (Δ9)

BLM_371 agatccattaccctgttatccctactctcgaggatct (Δ4)

BLM_377 agatccattaccctgttatccctactctcgaggatct (Δ9)

BLM_383 ccggagttgtcagatccattaccctgttatccctactctcgaggatctggcag (Δ40)

BLM_410 agatccattaccctgttatccctactctcgaggatct (Δ9)

BLM_466 agatccattaccctgttatccctactctcgaggatct (Δ14)

2) Middle deletion (60 bp – 1kb); 3 clones

BLM_67
gagaattaagagttactccgggccaaatggccggagttgtcagatccattaccctgttatccctactctcgaggatctggcagcgtcttcgct (Δ73)

BLM_2
gggtggacacagtcccccggcctggggagcctcgtgggagaattaagagttactccgggccaaatggccggagttgtcagatccattaccct
gttatccctactctcgaggatct (Δ108)

BLM_327
caaatggccggagttgtcagatccattaccctgttatccctactctcgaggatctggcagcgtcttcgctggggctccagggagctgctgctgggg
tggaagctctcacactctttctccacgtgccctttccagttccctgacatcgtggag (Δ131)

3) Large deletion (>1kb); 2 of 6 clones

BLM_176, BLM_243
ggcacagaggct--------------------
gtcagatccattaccctgttatccctactctcgaggatctggcagcgtcttcgctggggctccagggagctgct (Δ1518)

4) Insertion (1 bp); 2 clones

BLM_23, BLM_259 agatccattaccctgtttatccctactctcgaggatct (+1)

Supplement 4B

5) Rearrangement ; 5 clones

BLM_266 agatccagtaccctgttatccctactctcgaggatct (+1 / Δ9)

BLM_102
gctgcgcagttgtggatgtacctgtcgtctgctgggggcgtgcgggtggacacagtcccccggcctggggagcctcgtgggagaattaaga gttactccgggccaaatggccggagttgtcagatccattaccctgttatccctactctcgaggatctggcagcgtcttctccgagcccaaggca gcagctggggctccagggagctgc (Δ157 / +18 unknown)

BLM_432
gttgtcagatccattaccctgttatccctactctcgaggatctggcagcgtcttcgctggggctccagggagctgctgctggggtggaagctctca cactctttctccacgtgccctttccagttccctgacatcgtggagttctgcgaggccatggccaacgccgggaagaccgtaattgtggctgcact ggatgggaccttccagaggaaggtaaggcgtctgatccaggtctggagctgggattgaggagggcaagaggcttctggatgggcacaga gacaccagctctgggtgaccagggctcagccaccacagggttacggccgagctgctcaggccttggctgagccaagggactccatggtct gtgcagactgcgtgccatctgttgcggcaggtgctttgaattggcaaaggg (Δ97 / +129 inverted insertion of part of TK gene / Δ167)

BLM_85
ccgggccaaatggccggagttgtcagatccattaccc (sequence of part of pCBASce vector)tgttatccctactctcgagga (Δ30 / +1484 part of pCBASce vector)

BLM_172
aaatggccggagttgtcagatccattaccctgttatccctactctcgaggatctggcagcgtcttcgctggggctccagggagctgctgctggggt ggaagctctcacactctttctccacgtgccctttccagttccctgacatcgtggagttctgcgaggccatggccaacgccgggaagaccgtaattg tggctgcactggatgggaccttccagaggaaggtaaggcgtctgatccaggtctggagctgggattgaggagggcaagaggcttctggatgg gcacagagacaccagctctgggtgaccagggctcagccaccacagggttacggccgagctgctcaggccttggctgagccaagggactcc atggtctgtgcagactgcgtgccatctgttgcggcaggtgctttgaattggcaaagggacagagccgggcatggtgctctggggggttgggggaa ggactaaggtcagagcaaactctcctggcttcagtgcttgtgaatcagagggtttaaaagaaaaacccacctggtaaggtggctgagcgccctc tgtctttccatgggagcgcagccatttggggccatcctgaacctggtgccgctggccgagagcgtggtgaagctgacggcggtgtgcatggagt gcttccgggaagccgcctataccaagaggctcggcacagagaaggaggtagctccacctgccttccctgcaggccggcggggtgggggtat ggctctgcctccttcctgtcctggcccttcacccatcccctgtccctgcggccaggtcgaggtgattggggggagcagacaagtaccactccgtgtg tcggctctgctacttcaagaaggcctcaggccagcctgccgggccggacaacaaagagaactgcccagtgccaggaaagccaggggaag ccgtggctgccaggaagctctttgccccacagcagattctgcaatgcagccctgccaactgagggacctgcgagggccgcccgctcccttcct gccactgccgcctactggacgctgccctgcatgctgcccagccactccaggaggaagtcgggaggcgtggagggtgaccacaccttggcctt ctgggaactctcctttgtgtggctgccccacctgccgcatgctccctcctctcctacccactggtctgcttaaagcttccctctcagctgctgggtgac cttggcaatgacgatcgcccaggctggagctggccccgcttggtggcctgggatctggcacactccctctcct (Δ1178 / +14 unknown)

6) Recombination ; 2 clones

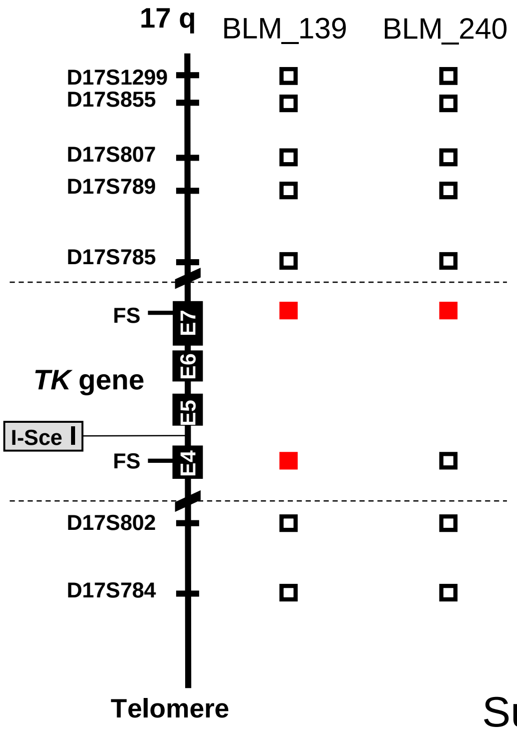

Supplement 4B (continued)

7) Translocation ; 1 clones

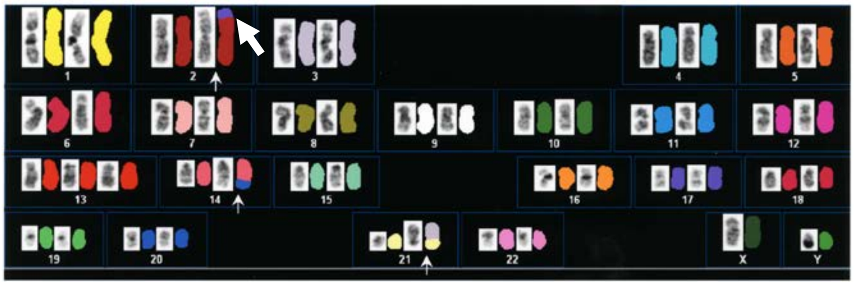

BLM_89

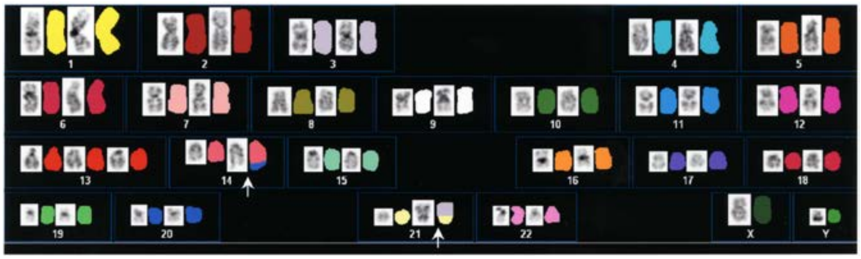

Parental BLM-TSCE5

Supplement 4B (continued)
